# The Prognostic Significance of CD44V6, CDH11, and **β**-Catenin Expression in Patients with Osteosarcoma

**DOI:** 10.1155/2013/496193

**Published:** 2013-07-18

**Authors:** Zhouming Deng, Guangfeng Niu, Lin Cai, Renxiong Wei, Xiaolei Zhao

**Affiliations:** ^1^Department of Orthopedics, Zhongnan Hospital of Wuhan University, No. 169 Donghu Road, Wuhan, Hubei Province 430071, China; ^2^Department of Spine, Shandong Provincial Hospital, Jinan 250021, China

## Abstract

This study aimed to examine the expression of and the relationship between CD44V6, CDH11, and **β**-catenin. The expression of these cell adhesion molecules was detected in 90 osteosarcoma and 20 osteochondroma specimens using immunohistochemistry. Associations between these parameters and clinicopathological data were also examined. The expression rates of CD44V6, CDH11, and **β**-catenin were 25.0% (5/20), 70.0% (14/20), and 20.0% (4/20) in osteochondroma specimens, respectively. Compared to osteochondromas, the proportions of expression of CD44V6 and **β**-catenin in osteosarcoma specimens increased to 65.6% (59/90) and 60.0% (54/90), respectively. However, the expression rate of CDH11 in osteosarcomas was reduced to 40.0% (36/90). The expression of these markers was significantly associated with metastasis and overall survival (*P* < 0.05). Survival analysis revealed that patients with increased expression of CD44V6 and **β**-catenin as well as decreased expression of CDH11 were correlated with a shorter survival time. Multivariate analysis indicated that clinical stage, metastasis status, and the expression of CD44V6, CDH11, and **β**-catenin were found to be associated with overall survival. Further, the expression of **β**-catenin and that of CD44V6 were positively correlated with each other. Thus, our results indicated abnormal expression of CD44V6, CDH11, and **β**-catenin in osteosarcomas and osteochondromas, which may provide important indicators for further research.

## 1. Introduction

Primary malignant tumors of bone are extremely rare neoplasm accounting for less than 0.2% of all cancers, although the true incidence is not easy to determine owing to the rarity of these tumors [1, 2]. Osteosarcoma is the most common form of primary bone cancer and accounts for approximately 19% of all malignant tumors of the bone. It is the third most common malignant tumor in teenagers [3]. Current treatments for osteosarcoma include surgical resection of both primary and pulmonary lesions, chemotherapy, and radiotherapy. Disease-free survival escalated from <20% prior to the introduction of effective chemotherapy to around 60% and overall survival to 60–70% [2, 4, 5]. However, the prognoses were unsatisfactory in patients with undetectable metastases at diagnosis [5]. In fact, only approximately 20–25% of newly diagnosed patients have clinically detectable metastatic disease at diagnosis [6]. At present, the ability to predict the prognosis of osteosarcomas is limited. Therefore, identifying prognostic markers of survival in osteosarcomas could be informative for selecting proper management. Traditional prognostic markers, such as gender, age, tumor location, disease-free interval, tumor doubling time, representation, and number of detectable pulmonary metastases, have had limited success in identifying those patients that need aggressive chemotherapy and those that do not [7]. More recently, the expression of certain biological molecules has been identified as potential prognostic markers for osteosarcoma metastasis, including the expression of CD44V6, CDH11, and *β*-catenin [7–9].

CD44 is a transmembrane glycoprotein, and some variant isoforms of CD44 (CD44V) are reportedly associated with increased invasion, metastasis, and poor prognosis [10–12], particularly the CD44 variant isoform V6 (CD44V6) [11–13]. It has been reported that CD44V6 can regulate the extracellular matrix, promote cell motility, and suppress tumor apoptosis. In fact, CD44V6 has been implicated in promoting tumor progression [11].

Cadherins comprise a major class of cell-cell adhesion molecules that interact with catenins to regulate adhesion and migration by Ca^2+^-dependent hemophilic interactions [14, 15]. These interactions are involved in maintenance of the tissue structure and morphogenesis as well as limiting cell movement and proliferation, thus resulting in tumor inhibition [16, 17]. Cadherin-11 (CDH11), also known as osteoblast cadherin, has been assigned a wide range of roles in relation to its influence on cancer cell behavior. It has been indicated that CDH11 expression may be useful as a prognostic marker of disease progression and survival in osteosarcomas [7].

The Wnt/*β*-catenin pathway has been implicated in the progression and pathogenesis of many forms of human cancer [18]. However, it has yet to be clarified whether Wnt/*β*-catenin signaling plays a role in osteosarcoma development. Several studies have reported that both nuclear and cytoplasmic *β*-catenin could be detected in osteosarcomas [19] or correlated with metastasis [9].

CD44V6, CDH11, and *β*-catenin belong to the family of cell adhesion molecules (CAM), which have been implicated in all aspects of cell growth, migration, and differentiation in vertebrate cells. Much research is focused on the relationship between CAM and cancer [7, 9, 11, 13–15, 19]. As part of ongoing efforts to understand the roles of and interactions between CD44V6, CDH11, and *β*-catenin in osteosarcomas, we report in this study our findings regarding the immunohistochemical expression and clinicopathological features of these biomarkers in human osteosarcomas. We have also analyzed the correlation of CD44V6, CDH11, and *β*-catenin with each other.

## 2. Materials and Methods

### 2.1. Patients and Samples

All patients or their guardians provided informed consent for participation in this study. This study was approved by the local research ethics committee (Zhongnan Hospital of Wuhan University). The exclusion criterion was inadequate follow-up data as well as preoperative chemotherapy or radiotherapy. Surgically resected osteosarcoma specimens were collected from 90 patients at Zhongnan Hospital of Wuhan University and Hubei Cancer Hospital, Hubei, China, between January 2000 and March 2008. The specimens were obtained from the primary site from 50 patients with metastases and 40 without metastases. No diagnostic biopsies were selected for this study. A total of 20 osteochondroma specimens were used as controls. Formalin-fixed, paraffin-embedded blocks were retrieved from the Department of Pathology. All the samples were evaluated for diagnosis by 3 experienced pathologists. Of the 90 patients, 53 were male and 37 were female. The median age of the patients was 18.3 years, with a range of 8–58 years. Clinical stage was graded according to the standard of Enneking et al. [20]; there were two cases of stage IB, 24 cases of stage IIA, 39 cases of stage IIB, and 25 cases of stage III. For the 20 osteochondroma patients, 12 were male and 8 were female, with an average age of 60.4 years ranging from 21 to 78 years. 

### 2.2. Immunohistochemistry

The tissues were fixed in 10% buffered formalin and processed using standard procedures. The sections (4-5 *μ*m) obtained from representative tissue sample blocks were heated at 60°C for 20 min, deparaffinized with xylene, and rehydrated using graded ethanol. After quenching endogenous peroxidase activity with 3% hydrogen peroxide in methanol for 10 min, the slides were microwaved in 10 mmol/L sodium citrate (pH 6.0) for 15 min to retrieve the antigen. The elimination of nonspecific staining was performed with diluted normal goat serum. The sections were then incubated at 37°C for 1 h with primary antibodies against CD44V6 (ZSGB-BIO, China), CDH11 (RD Biosciences, USA), and *β*-catenin (ZSGB-BIO, China). Following three successive rinses with phosphate buffered saline (PBS), they were further incubated with secondary antibody (Maixin Bio, China) for 40 min at 37°C. The sections were allowed to develop using a DAB color kit (ZSGB-BIO, China) for 5 min and then were counterstained with hematoxylin. For each protein, the immunostaining in patient samples was compared with osteochondroma as a normal control, while negative controls were obtained by omitting the primary antibody, substituted by PBS. 

### 2.3. Evaluation of Immunohistochemistry

The “immunohistochemical score” (IHS) was calculated based on previously published research [21]. Immunoreactivity was evaluated prior to the collection of the patient identity and clinical information. The IHC classification of positivity was scored as follows: (1) ≤25% of cells staining positively; (2) 26%–50% of cells staining positively; (3) 51%–75% of cells staining positively; (4) 76%–100% of cells staining positively. The intensity of the immunoexpression was rated as negative (0), weak (1), moderate (2), or strong (3). A consensus was achieved by three of the authors in all cases. The final IHS was obtained by multiplying the score of extent and intensity. The IHS of each specimen was categorized into four groups: −, (0–2); +, (3–5); ++, (6–8); +++, (9–12). Scores of 0–5 were designated as low expression, while scores of 6–12 were designated as expression.

### 2.4. Statistical Analysis

The whole statistical analysis was performed with SPSS version 18.0 software (SPSS Inc, Chicago, USA). The correlation between antigen expression and the clinicopathological parameters was assessed by chi-square test or Kruskal-Wallis test when chi-square test was not suitable. Survival rates were estimated by Kaplan-Meier statistics and survival curves were compared by using the Log-rank test. A Cox regression method was used for multivariate analysis. In this paper, we only investigated the relation between expressions of the three markers with overall survival; the metastasis-free survival was not studied. Survival was calculated from the time of the primary operation. An observation was censored at the last followup if the patient was alive or had died of a cause other than osteosarcoma. The correlation between CD44V6, CDH11, and *β*-catenin was assessed by Spearman's correlation analysis. A *P* value of less than 0.05 was considered statistically significant for all tests.

## 3. Results

### 3.1. Expression of CD44V6, CDH11, and *β*-Catenin and Their Correlations with Clinicopathological Features of Patients with Osteosarcoma

**Figure 1:**
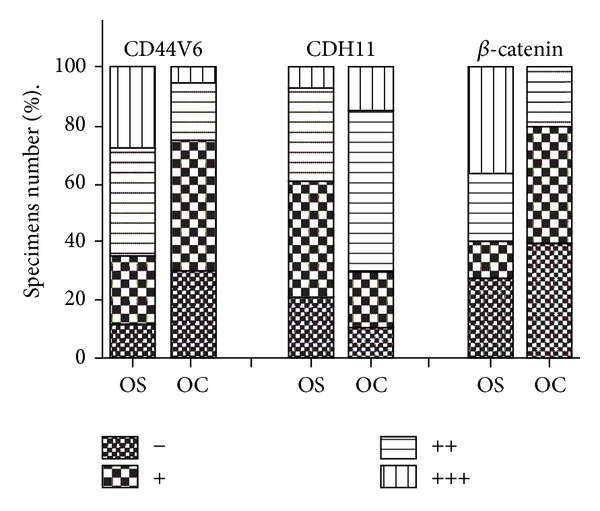
The immunohistochemical expression of CD44V6, CDH11, and *β*-catenin in osteosarcoma and osteochondroma samples.

**Figure 2:**
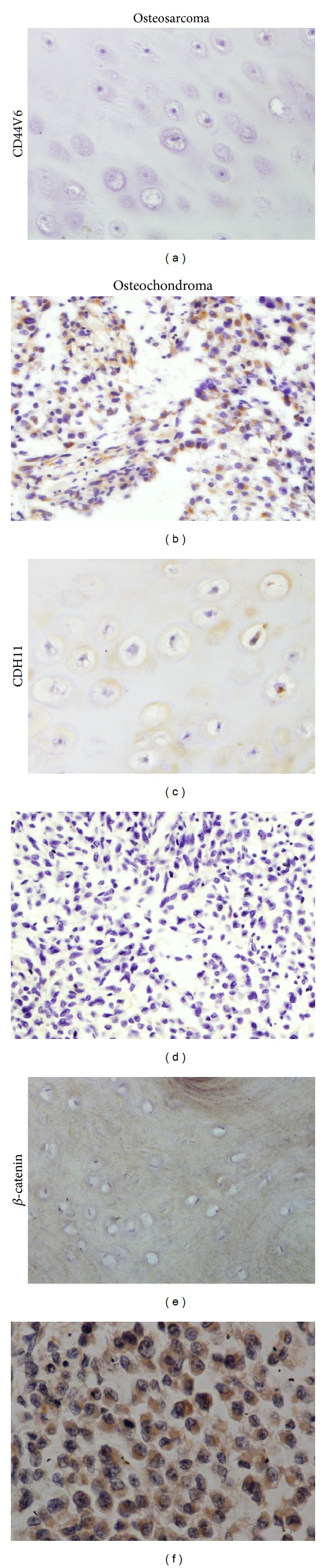
Representative expression of CD44V6, CDH11, and *β*-catenin in osteosarcoma and osteochondroma samples.

The different expression levels of the three markers in osteosarcoma and osteochondroma are depicted in Figure 1 (Table 1). Of the osteochondroma cases, diffuse expression of CD44V6 was observed in 5 cases (25.0%), CDH11 in 14 cases (70.0%), and *β*-catenin in 4 cases (20.0%) (Table 1). In contrast, the proportions of expression of CD44V6 and *β*-catenin in osteosarcoma cases were increased to 65.6% (59/90) and 60.0% (54/90), respectively. However, the rate of expression of CDH11 in osteosarcomas was reduced compared to osteochondromas to 40.0% (36/90). Representative staining in osteosarcoma specimens is depicted in Figure 2. The chi-square test demonstrated that the difference in expression of these three parameters between osteosarcomas and osteochondromas demonstrated statistical significance (*P* < 0.05, Table 1). 

Total CD44V6, CDH11, and *β*-catenin protein staining was associated with metastasis (*P* < 0.05). The expression of CD44V6, CDH11, and *β*-catenin was not significantly associated with age, gender, tumor site, and histological subtype (Table 2).

### 3.2. Prognostic Values of CD44V6, CDH11 and *β*-Catenin Expressions

**Figure 3:**
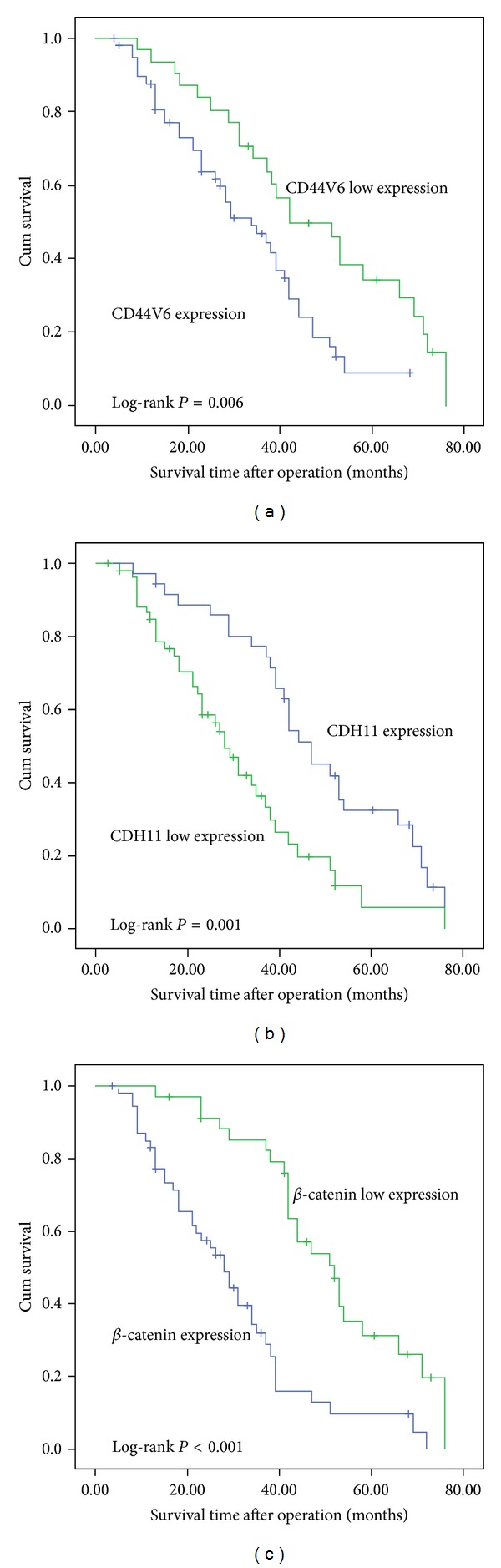
Postoperative survival curves for patients, according to the expression of CD44V6, CDH11, and *β*-catenin, respectively.

**Figure 4:**
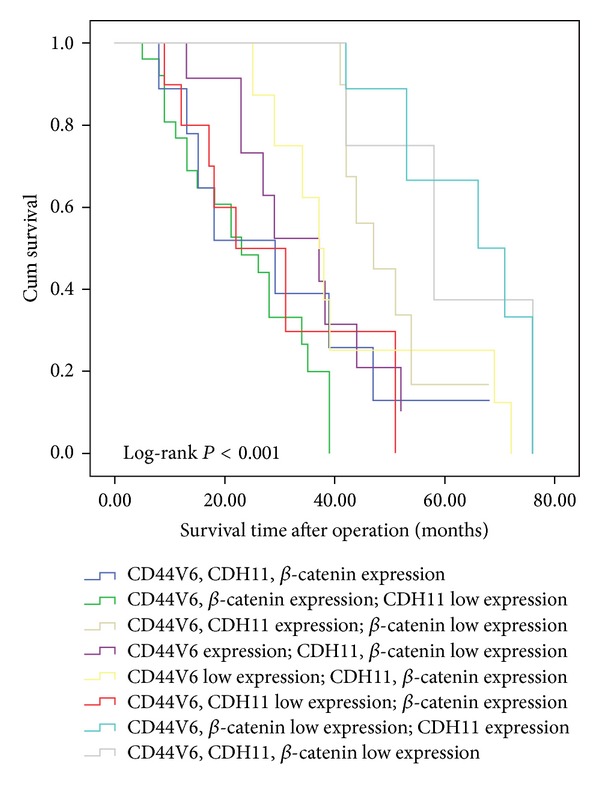
Postoperative survival curves for patients, according to the conjoint analysis of CD44V6, CDH11, and *β*-catenin expression.

Survival analysis was conducted using Kaplan-Meier curves for overall survival and univariate analysis. All three potential surrogate endpoints proved to alter the survival probability with *P* values below 0.01 (Log-rank test, Figure 3). Based on the expression of all three proteins, the patients were reassigned to eight groups. The survival analysis revealed that a shorter survival time was correlated with patients who demonstrated expression of CD44V6 and *β*-catenin as well as low expression of CDH11. On the other hand, low expression of CD44V6 and *β*-catenin as well as expression of CDH11 indicated better survival (*P* < 0.05, Figure 4).

Proceeding to multivariate analysis, Cox multivariate analysis showed that the expression of the three markers, clinical stage, and metastasis status remained significantly associated with overall survival, whereas age and gender were not (Table 3).

### 3.3. The Correlation of CD44V6, CDH11, and *β*-Catenin in Osteosarcoma

To determine whether these CAMs were associated with each other in osteosarcomas, we performed correlative analysis. The results revealed that CD44V6 expression was positively correlated with the expression of *β*-catenin (*r* = 0.768, *P* < 0.001, Table 4). 

## 4. Discussion

Osteosarcoma is the most frequent primary cancer of bone (incidence: 0.2–0.3/100 000/year). The incidence is higher in adolescents (0.8–1.1/100 000/year at age 15–19), where it accounts for >10% of all solid cancers [2]. Currently, there is a lack of understanding of the molecular mechanisms leading to the development and progression of osteosarcomas. Regardless of intensifying and modifying chemotherapy, limited improvements to survival of osteosarcoma patients have been achieved over the past 20 years [22]. The development of metastasis to the lungs represents the most common cause of death in osteosarcoma patients. Despite growing evidence implicating roles for particular molecular markers and pathways in the initiation and progression of osteosarcoma, their clinical significance remains debatable. Recently, evidence has revealed that a phenomenon called “chromothripsis” can trigger between tens and hundreds of genomic rearrangements in multiple cancer samples, promoting the development of cancer. The complex genomic rearrangement, with frequent copy number changes, confined to localized genomic regions rapidly alternating between usually no more than three different states, occurred in at least 2-3% of all cancers. The genomic features imply chromosome breaks occur in a single catastrophic event rather than as a cumulative acquisition of mutations. The “chromothripsis” was especially common in osteosarcomas (more than 30%), which was suggested as a critical event in the conversion of a normal cell to a cancerous cell [23]. Another study revealed a notable association between TP53 mutation and “chromothripsis” [24]. The metastatic cascade remains a complex process, and the CAMs play an important role in the first step of tumor metastasis [25, 26]. To confirm our hypothesis that CD44V6, CDH11, and *β*-catenin are potential tumor markers, their protein level and relationship with prognosis were analyzed using immunochemistry.

Our results revealed that CD44V6 was expressed in 25.0% of the collected samples of osteochondroma, whereas, CD44V6 was expressed in 65.6% of osteosarcoma samples. The findings in our study are consistent with previous research demonstrating that the expression of CD44V6 was correlated with metastasis and poor prognosis in patients with osteosarcoma [8]. It was also reported that the higher expression of CD44V6 correlated with metastasis and poorer survival in patients with other tumors (i.e., pancreatic cancer, colorectal cancer, ovarian cancer, bladder cancer, lung cancer, and glioblastoma multiforme) [27–32]. In addition, Fan et al. [33] performed a meta-analysis, in which CD44V6 positive cells (OR = 0.36, *P* = 0.02) were significantly associated with poor overall survival in patients with colorectal cancer. The mechanism of CD44V6 promoting the metastasis of cancer may be attributed to its interactions with various components of the extracellular matrix and its involvement in cell adhesion and critical signaling pathways, for example, Ras and Akt [34, 35]. Nakajima et al. [36] suggested that CD44V6 could be an oncofetal protein in the bone tissue, which could be expressed in the osteosarcoma when it metastasizes. Furthermore, phase I clinical trials of CD44V6 antibodies that were either radiolabeled or covalently linked to a toxin were investigated in patients affected by head and neck squamous cell carcinomas, with the outcome of the trial providing promising results [37]. These results reinforce our findings. However, there are other inconsistent reports of the implications of CD44V6 expression in cancer. Yang et al. [38] found that decreased CD44V6 expression promoted the recurrence and carcinogenesis of parotid pleomorphic adenoma. Spafford et al. [39] reported that increased CD44V6 expression was consistent with longer survival (*P* < 0.02) of patients with laryngeal squamous cell carcinoma. In addition, the association of CD44V6 expression with malignancy and survival could not be confirmed in several studies investigating osteosarcomas as well as other tumors [40–43]. Therefore, the suitability of CD44V6 expression to be used as a prognostic marker remains a matter of debate.

In this study, a significant correlation was found between CDH11 expression and patient survival, which is consistent with a previous study [7]. Several studies have suggested that CDH11 displays tumor suppressor properties in osteosarcomas and other tumors [44–48]. The loss or decrease of CDH11 expression plays an important role in osteosarcoma metastasis [47]. Kashima et al. [48] have found that osteosarcoma metastasis can be prevented by restoration of CDH11 expression using an *in vivo* metastasis assay. It has been suggested that tumor-promoting inflammation and antitumor immunity coexist at different points along the path of tumor progression [49], and a recent report has demonstrated that CDH11 was a key mediator of fibroblast inflammation [50]. Consequently, it appears that CDH11 may be involved in osteosarcoma invasion and metastasis through a potential link between inflammation and tumor development. 

*β*-catenin is an intracellular protein with two important cellular functions: The cadherin-bound *β*-catenin is required for cell adhesion, while nuclear *β*-catenin transmits extracellular-initiated Wnt signals to the nucleus. *β*-catenin is involved with differentiation and proliferation of cells in a wide variety of tissues, and the Wnt signaling pathway has emerged as an essential pathway in skeletal development and disease [51]. Several studies have indicated that active Wnt signaling is conducive to osteosarcoma progression based on cytoplasmic or membranous *β*-catenin staining [9, 19, 52]. However, this may not prove the role of active Wnt signaling owing to the dual role of *β*-catenin. Considering this pivotal role of *β*-catenin, we also analyzed *β*-catenin expression in our study. Cytoplasmic immunostaining was observed in most cases of osteosarcoma (74/90), and we observed that the expression of *β*-catenin was significantly increased in osteosarcomas compared to osteochondromas. Furthermore, low expression of *β*-catenin was correlated with longer survival time. Several studies have revealed that positive cytoplasmic *β*-catenin expression was associated with the development of metastasis both *in vivo* and *in vitro* [9, 52–54]. Although the deregulation of *β*-catenin is thought to play an important role in oncogenesis of osteosarcomas, the role of Wnt signaling in osteosarcomas remains controversial [18, 22, 53, 55, 56]. Recently, a study has indicated that *β*-catenin may interact with other proteins, such as NF-kappa B, during oncogenesis [57]. Further studies using new immunohistochemical markers of the Wnt signaling pathway are needed to investigate the potential role of this pathway in osteosarcoma pathogenesis.

We also found that the expression of CD44V6 was significantly positively correlated with that of *β*-catenin (*P* < 0.001). The correlation between CD44V6 and *β*-catenin was concordant with a previous study, which showed that CD44 overexpression (CD44S and CD44V6) was associated with activation of *β*-catenin, suggesting CD44 is one of the target genes of *β*-catenin [58]. 

An obvious shortcoming of our study is that immunohistochemistry is, at best, a semiquantitative technique. The results should be considered exploratory and caution should be taken in interpreting data. Moreover, as for other prognostic factors, the functions of these molecular markers may vary depending on the tissue context. These results may not be applicable to other tumor types. Another limitation of our study is that osteochondromas were used as a control due to limited conditions, and therefore the results may not be as convincing as a comparison with normal bone specimens. Further studies using normal bone tissue as controls are needed. Despite these limitations, noteworthy results were acquired. The expression of CD44V6, CDH11, and *β*-catenin could be a potential prognostic indicator, especially conjoint analysis of the three markers. The relevant molecular mechanisms require further investigation. Considering that osteosarcoma is a very rare disease, this emphasizes the need for multi-institutional collaboration to identify and validate new biomarkers. 

## Figures and Tables

**Table 1:** Expression of CD44V6, CDH11, and *β*-catenin in osteosarcomas and osteochondromas.

		CD44V6 expression			CDH11 expression			*β*-catenin expression		
	Cases	Expression	Low expression	*P *	Expression	Low expression	*P *	Expression	Low expression	*P *
OS	90	59 (65.6%)	31 (34.4%)	0.0009	36 (40.0%)	54 (60.0%)	0.042	54 (60.0%)	36 (40.0%)	0.001
OC	20	5 (25.0%)	15 (75.0%)		14 (70.0%)	6 (30.0%)		4 (20.0%)	16 (80.0%)	

OS: osteosarcoma, OC: osteochondroma.

**Table 2:** Correlation between the expression of CD44V6, CDH11, and *β*-catenin and clinicopathological data.

Clinical features	CD44V6 expression			CDH11 expression			*β*-catenin expression		
	Expression	Low expression	*P *	Expression	Low expression	*P *	Expression	Low expression	*P *
Age			0.168			0.418			0.928
≤50	18	14		11	21		19	13	
>50	41	17		25	33		35	23	
Gender			0.432			0.600			0.097
Male	33	20		20	33		28	25	
Female	26	11		16	21		26	11	
Histological classification			0.179			0.382			0.891
Osteoblastic	32	11		18	25		27	16	
Chondroblastic	11	7		9	9		11	7	
Fibroblastic	8	5		2	11		8	5	
Telangiectatic	4	7		5	6		5	6	
Mixed	4	1		2	3		3	2	
Primary site			0.495			0.667			0.367
Femur	28	15		17	26		23	20	
Tibia	12	9		11	10		16	5	
Humerus	8	5		4	9		8	5	
Fibula	1	1		1	1		2	0	
Ilium	5	1		1	5		3	3	
Other	5	0		2	3		2	3	
Metastasis			0.02*			0.009*			0.002*
Yes	38	12		14	36		37	13	
No	21	19		22	18		17	23	

*P < 0.05.

**Table 3:** Multivariate survival analysis of overall survival in patients with osteosarcoma.

Variables	*P *	RR	95% CI for RR
Age	0.321	1.019	0.982–1.057
Gender	0.221	0.749	0.471–1.190
Metastasis status	0.019	2.067	1.129–3.784
Clinical stage	0.000	2.288	1.559–3.360
CD44V6 expression	0.042	0.586	0.350–0.982
CDH11 expression	0.010	1.934	1.172–3.191
*β*-catenin expression	0.002	0.425	0.249–0.727

**Table 4:** The correlation analysis between expression of CD44V6, CDH11, and *β*-catenin.

	CD44V6	CDH11	*β*-catenin
CD44V6		*rs* = 0.200, *P* = 0.059	*rs* = 0.768, *P* < 0.001*
CDH11	*rs* = 0.200, *P* = 0.059		*rs* = −0.231, *P* = 0.029*
*β*-catenin	*rs* = 0.768, *P* < 0.001*	*rs* = −0.231, *P* = 0.029*	

rs: Spearman's rank correlation coefficient, two-tailed significances, *P < 0.05.
